# Renal denervation using externally delivered focused ultrasound: summary of clinical experience to date and validation of supporting computational simulations

**DOI:** 10.1186/2050-5736-3-S1-O68

**Published:** 2015-06-30

**Authors:** Michael Gertner, Francesco Crura, Jimin Zhang

**Affiliations:** 1 Kona Medical, Bellevue, Washington, United States

## Background/introduction

The Kona Medical Surround Sound™ System is a fully non-invasive system for renal denervation which employs ultrasound based imaging for targeting and tracking as well as a phased array therapeutic ultrasound transducer. To date, 69 patients have been treated under three different protocols (WAVE I, WAVE II, and WAVE III).

Follow up ranges from 3 months to over 2 years. A subset of 18 patients (WAVE II) was chosen for detailed analysis using numerical simulation. Aims: To evaluate safety, feasibility, and a specific dose strategy using a computational model in a Virtual Acoustic Patient (VAP) model and validate these simulations with human data.

## Methods

A 3D human body acoustic and thermal simulation models has been developed. The model utilizes the cross sectional imaging from MRI or CT of the treated patients and translates these images into 3D acoustic models. Subsequently, the key parameters and variables that influence the efficacy and safety of the high intensity therapeutic ultrasound (HITU) treatment were incorporated into the model. Specific anatomic regions which relate to safety and efficacy were highlighted in the model.

## Results and conclusions

The simulations in the virtual acoustic patient models demonstrated the following:

1. The therapeutic dose creates a thermal lesion around the renal artery.

2. There are no significant pre-focal or post-focal regions of unwanted thermal or mechanical tissue damage in vital organs including the kidneys, spine, and bowel.

3. The maximum peak temperature used in the clinical trials is below the thermally-induced cavitation using the most extreme clinical conditions and absorption coefficients.

4. The contribution of non-linear content in the acoustic waveform is minimal and is highly unlikely to result in any significant unintended mechanical biological effects such as cavitation.

5. There is potential for unwanted heating starting from the fat/muscle interface and inside fat layer using the extremes of absorption coefficients and patients depths.

Clinical data in 58 patients who have reached 6 mos follow-up show an average blood pressure drop of 24/10 from baseline and an acceptable safety profile.

Conclusions: Computational ultrasound modeling and simulation developed using the virtual acoustic patient models provides a powerful tool for evaluating and validating the mechanical and thermal effects of treatment protocol used in the clinical for renal denervation. Clinical data correlates well with the simulation and provides validating evidence that the simulation can be predictive of clinical results.

